# Supplementary material for: HIVprotI: an integrated web based platform for prediction and design of HIV proteins inhibitors
Source: J Cheminform. 2018 Mar 9;10:12. doi: 10.1186/s13321-018-0266-y (PMC5845081; doi:10.1186/s13321-018-0266-y)
Supplement: Supplementary file 2 — Additional file 2. Source code of HIVProtI web server. [file 13321_2018_266_MOESM2_ESM.zip › HIVprotI_Source-code/hivprotI/weka-3-6-6/doc/weka/core/Tag.html]

Tag


---


|  |  |  |
| --- | --- | --- |
| **Overview** \| **Package** \| **Class** \| **Tree** \| **Deprecated** \| **Index** \| **Help** \| **Weka's home** | |  |
| **PREV CLASS**   **NEXT CLASS** | **FRAMES**    **NO FRAMES**     **All Classes** |  |
| SUMMARY: NESTED \| FIELD \| CONSTR \| METHOD | DETAIL: FIELD \| CONSTR \| METHOD |  |


---


## weka.core Class Tag

```
java.lang.Object
  weka.core.Tag
```

**All Implemented Interfaces:**: java.io.Serializable, RevisionHandler

---

``` public class Tag extends java.lang.Object implements java.io.Serializable, RevisionHandler ```

A `Tag` simply associates a numeric ID with a String description.

**Version:**
:   $Revision: 1.13 $

**Author:**
:   Len Trigg

**See Also:**: Serialized Form

---

**Constructor Summary**

`Tag()`
  
          Creates a new default Tag

`Tag(int ident,
java.lang.String readable)`
  
          Creates a new `Tag` instance.

`Tag(int ident,
java.lang.String identStr,
java.lang.String readable)`
  
          Creates a new `Tag` instance.

`Tag(int ident,
java.lang.String identStr,
java.lang.String readable,
boolean upperCase)`


| **Method Summary** | |
| --- | --- |
| `int` | `getID()`             Gets the numeric ID of the Tag. |
| `java.lang.String` | `getIDStr()`             Gets the string ID of the Tag. |
| `java.lang.String` | `getReadable()`             Gets the string description of the Tag. |
| `java.lang.String` | `getRevision()`             Returns the revision string. |
| `void` | `setID(int id)`             Sets the numeric ID of the Tag. |
| `void` | `setIDStr(java.lang.String str)`             Sets the string ID of the Tag. |
| `void` | `setReadable(java.lang.String r)`             Sets the string description of the Tag. |
| `static java.lang.String` | `toOptionList(Tag[] tags)`             returns a list that can be used in the listOption methods to list all the available ID strings, e.g.: <0\|1\|2> or <what\|ever> |
| `static java.lang.String` | `toOptionSynopsis(Tag[] tags)`             returns a string that can be used in the listOption methods to list all the available options, i.e., "\t\tID = Text\n" for each option |
| `java.lang.String` | `toString()`             returns the IDStr |

**Methods inherited from class java.lang.Object**

`equals, getClass, hashCode, notify, notifyAll, wait, wait, wait`

**Constructor Detail**

### Tag

```
public Tag()
```

:   Creates a new default Tag

---


### Tag

```
public Tag(int ident,
           java.lang.String readable)
```

:   Creates a new `Tag` instance.

    **Parameters:**: `ident` - the ID for the new Tag.: `readable` - the description for the new Tag.

---


### Tag

```
public Tag(int ident,
           java.lang.String identStr,
           java.lang.String readable)
```

:   Creates a new `Tag` instance.

    **Parameters:**: `ident` - the ID for the new Tag.: `identStr` - the ID string for the new Tag (case-insensitive).: `readable` - the description for the new Tag.

---


### Tag

```
public Tag(int ident,
           java.lang.String identStr,
           java.lang.String readable,
           boolean upperCase)
```


**Method Detail**

### getID

```
public int getID()
```

:   Gets the numeric ID of the Tag.

    :   **Returns:**: the ID of the Tag.

---


### setID

```
public void setID(int id)
```

:   Sets the numeric ID of the Tag.

    :   **Parameters:**: `id` - the ID of the Tag.

---


### getIDStr

```
public java.lang.String getIDStr()
```

:   Gets the string ID of the Tag.

    :   **Returns:**: the string ID of the Tag.

---


### setIDStr

```
public void setIDStr(java.lang.String str)
```

:   Sets the string ID of the Tag.

    :   **Parameters:**: `str` - the string ID of the Tag.

---


### getReadable

```
public java.lang.String getReadable()
```

:   Gets the string description of the Tag.

    :   **Returns:**: the description of the Tag.

---


### setReadable

```
public void setReadable(java.lang.String r)
```

:   Sets the string description of the Tag.

    :   **Parameters:**: `r` - the description of the Tag.

---


### toString

```
public java.lang.String toString()
```

:   returns the IDStr

    :   **Overrides:**: `toString` in class `java.lang.Object`
    :   **Returns:**: the IDStr

---


### toOptionList

```
public static java.lang.String toOptionList(Tag[] tags)
```

:   returns a list that can be used in the listOption methods to list all
    the available ID strings, e.g.: <0|1|2> or <what|ever>

    :   **Parameters:**: `tags` - the tags to create the list for **Returns:**: a list of all ID strings

---


### toOptionSynopsis

```
public static java.lang.String toOptionSynopsis(Tag[] tags)
```

:   returns a string that can be used in the listOption methods to list all
    the available options, i.e., "\t\tID = Text\n" for each option

    :   **Parameters:**: `tags` - the tags to create the string for **Returns:**: a string explaining the tags

---


### getRevision

```
public java.lang.String getRevision()
```

:   Returns the revision string.

    :   **Specified by:**: `getRevision` in interface `RevisionHandler`
    :   **Returns:**: the revision


---


|  |  |  |
| --- | --- | --- |
| **Overview** \| **Package** \| **Class** \| **Tree** \| **Deprecated** \| **Index** \| **Help** \| **Weka's home** | |  |
| **PREV CLASS**   **NEXT CLASS** | **FRAMES**    **NO FRAMES**     **All Classes** |  |
| SUMMARY: NESTED \| FIELD \| CONSTR \| METHOD | DETAIL: FIELD \| CONSTR \| METHOD |  |


---
